# Influence of theta-burst transcranial magnetic stimulation over the dorsolateral prefrontal cortex on emotion processing in healthy volunteers

**DOI:** 10.3758/s13415-020-00834-0

**Published:** 2020-09-30

**Authors:** Ana Dumitru, Lorenzo Rocchi, Fedal Saini, John C. Rothwell, Jonathan P. Roiser, Anthony S. David, Raphaelle M. Richieri, Gemma Lewis, Glyn Lewis

**Affiliations:** 1 Division of Psychiatry, University College London, Maple House, 6th Floor, 149 Tottenham Court Rd, Fitzrovia, London, W1T 7BN UK; 2 Institute of Neurology, Department of Clinical and Movement Neurosciences, University College London, London, UK; 3 Institute of Cognitive Neuroscience, University College London, London, UK; 4 Department of Psychiatry, Aix-Marseille University, Marseille, France

**Keywords:** Theta-burst stimulation (TBS), Dorsolateral prefrontal cortex (DLPFC), Emotional processing

## Abstract

Repetitive transcranial magnetic stimulation is a potential treatment option for depression, with the newer intermittent theta-burst stimulation (iTBS) protocols providing brief intervention. However, their mechanism of action remains unclear. We investigated the hypothesis that iTBS influences brain circuits involved in emotion processing that are also affected by antidepressants. We predicted that iTBS would lead to changes in performance on emotion-processing tasks. We investigated the effects of intermittent TBS (iTBS) over the left dorsolateral prefrontal cortex (DLPFC) on the processing of emotional information (word recall and categorization, facial emotion recognition, and decision-making) in 28 healthy volunteers by contrasting these effects with those of sham stimulation. Each volunteer received iTBS and sham stimulation in a blinded crossover design and completed the emotion-processing tasks before and after stimulation. Compared to sham stimulation, iTBS increased positive affective processing for word recall, yet had an unexpected effect on facial emotion recognition for happy and sad faces. There was no evidence of an effect on decision-making or word categorization. We found support for our hypothesis that iTBS influences emotion processing, though some changes were not in the expected direction. These findings suggest a possible common mechanism of action between iTBS and antidepressants, and a complex neural circuitry involved in emotion processing that could potentially be tapped into via brain stimulation. Future research should investigate the neural correlates of emotion processing more closely to inform future iTBS protocols.

## Introduction

Depression is the leading cause of disability worldwide (World Health Organization, 2017), making the need for effective treatments options important (Cuijpers et al., 2017). Due to a significant proportion of patients failing to respond to psychiatric medication or therapy (Amick et al., 2015; Barth et al., 2013; Cipriani et al., 2018), exciting brain-stimulation methods have evolved as a potential therapeutic tool. One such method, repetitive transcranial magnetic stimulation (rTMS), induces a current flow within cortical brain regions by producing an alternating electromagnetic field delivered in brief, but powerful pulses – 1.5–3 Tesla strong (George & Post, 2011). When compared to single pulses, repeated pulses have longer-lasting effects, believed to induce cortical plasticity (Klomjai et al., 2015); such effects depend on stimulation parameters (i.e. frequency of pulses, number of trains, duration of treatment, intensity of stimulation, etc.) (Ridding & Rothwell, 2007). Due to its neuromodulatory effects on cortical activity (Wassermann & Zimmermann, 2012), rTMS has received particular attention in the treatment of depression. An antidepressant effect of 10 Hz rTMS over the prefrontal cortex (PFC) was first reported by Pascual-Leone et al. (1996), with subsequent studies providing robust evidence for short-term effectiveness (Eshel et al., 2017; Fitzgerald et al., 2016; George, 1998; Weissman et al., 2017). Despite the multitude of studies conducted in this field, we still do not know how rTMS affects different brain regions, which in turn affect behaviour.

The dorsolateral PFC (DLPFC) is a frontal brain area that has been targeted in rTMS studies involving emotion processing and depressive symptoms (Padberg & George, 2009; Baeken & De Raedt, 2011). The DLPFC is involved in processing of emotions, specifically top-down emotional control (Cacioppo et al., 1993; Dolcos et al., 2004; Zwanzger et al., 2014), and is more broadly linked to working memory and cognitive control, processes that can become impaired during depression (Fales et al., 2008; Grimm et al., 2008; Koenigs & Grafman, 2009; Rock et al., 2014). Studies involving the 10 Hz rTMS over the DLPFC have provided interesting insight, in both depressed and healthy individuals, regarding its positive influence on working memory and emotion processing (Weigand et al., 2013; Guo et al., 2019). The beneficial effects of rTMS over the DLPFC are believed to stem from the connections of the PFC with the limbic system, particularly the hippocampus and amygdala (Groenewegen & Uylings, 2000). Moreover, researchers have argued that high-frequency rTMS to the DLPFC could help regulate stress responses through connections with the hypothalamic-pituitary axis, which can become dysregulated during depression (Baeken & De Raedt, 2011). More recent studies found rTMS modulates functional connectivity between the DLPFC and other limbic regions such as the subgenual anterior cingulate cortex (Kito et al., 2017).

Previous brain stimulation research involving transcranial direct current stimulation (tDCS), as well as rTMS protocols, has indicated that transcranial brain stimulation over the DLPFC improves emotion processing in both healthy and patient populations, by inhibiting negative bias and increasing excitability for positive stimuli (Brunoni et al., 2014; Guo et al., 2019; Ironside, O’Shea, Cowen, & Harmer, 2016; Nitsche et al., 2012). Authors argue that stimulation methods such as rTMS, if applied over the left PFC, can modulate affective processing (Choi, Scott, & Lim, 2016; Schutter & van Honk, 2005). For instance, a study performed in a schizophrenic cohort showed that rTMS improved facial affect recognition (Wölwer et al.*,* 2014). In another study on healthy participants, Skrdlantová et al. (2005) talked about localization of word memory to the left PFC and accessing it via rTMS. Stimulation of this area seems to improve cognitive control and decision-making as well (Li et al.*,* 2017; Philiastides et al.*,* 2011). Moreover, rTMS over the left DLPFC appears to improve processing and especially memory retrieval for affective information (Balconi & Ferrari, 2012b; Balconi & Cobelli, 2014). Studies in which the left DLPFC was stimulated have seen changes in facial-affect processing (De Raedt et al.*,* 2010; Mondino, Thiffault, & Fecteau, 2015; Moulier et al.*,* 2016).

Intermittent theta-burst stimulation (iTBS) involves high-frequency trains of stimulation and is based on the naturally occurring theta-wave patterns exhibited by neurons (Huang et al., 2005). Non-inferior in effectiveness, but with a shorter duration compared to 10-Hz rTMS, iTBS could allow for more people to be treated daily (Blumberger et al., 2018). Despite these promising clinical results, the mechanism through which iTBS treats depressive symptoms remains poorly understood (Cramer et al., 2011; Suppa et al., 2016).

We propose that iTBS over the DLPFC would have a similar cognitive effect to that of an antidepressant. A possible candidate mechanism for the antidepressant effects of iTBS is through emotion processing. Numerous studies using objective neuropsychological measures have found that people with depression show abnormalities in their attention, memory, and learning when processing emotional information, and this is altered by antidepressant medication (Lewis et al., 2017; Roiser, Elliott, & Sahakian, 2011; Roiser & Sahakian, 2016). Harmer et al. (2009) have suggested that antidepressants work via a rapid change in emotion processing that then leads to a slower improvement in mood. Lower scores on depression scales were robustly associated with more positive emotion processing in a large sample (Lewis et al., 2017), while antidepressants appeared to increase positive affective processing in healthy and depressed people alike (Harmer et al., 2009). A study by Bone et al. (2019) looked at the variation in happy and sad facial recognition in a depressive population, finding no association between hit rates and depressive symptoms, and some evidence of an association for the misclassification of ambiguous happy faces (happy false alarms) and reduced depression scores.

In this study, our aim was to investigate the effects of iTBS over the left DLPFC on emotion processing in healthy participants in order to test our hypothesis concerning the mechanism of action of iTBS. Based on the previous studies discussed so far, which maintain that stimulation of the DLPFC increases positive affect and modulates emotion processing, we expected that iTBS over the left DLPFC would improve performance on three affective processing tasks, targeting three important domains of mood-related information processing: emotional memory and categorization for words (Emotional Categorization Word Task), emotion recognition in faces (Emotional Recognition Task), and decision-making (Cambridge Gambling Task). We predicted that iTBS would increase positive affective processing as measured by these tasks (i.e. greater recall and improved categorization of positive words, greater hits and false alarms for happy faces, and greater risk adjustment for win and loss conditions, respectively). We chose these tasks as they have been previously used in similar studies or they are relevant in the context of DLPFC circuitry (i.e. Balconi & Ferrari, 2012a; Bland et al., 2016; Lewis et al., 2017; Yazdi et al., 2019).

## Methods and materials

### Participants

Data were collected from 28 healthy, right-handed volunteers, with normal or corrected-to-normal vision (mean age 27 years, age range 19–44 years, SD = 6.52; 17 males), recruited via their response to an advert posted through the University College London (UCL) Institute of Cognitive Neuroscience database. Exclusion criteria consisted of any history of neurological or psychiatric illness – including stroke, seizure, or epilepsy, any implanted devices such as pacemakers, any use of psychotropic medication, and any significant sleep deprivation. Furthermore, participants were asked to refrain from consuming alcohol or caffeine prior to the study session (Silvanto & Pascual-Leone, 2008).

This research study was approved by the UCL Research Ethics Committee and was conducted in accordance with the World Medical Association Declaration of Helsinki (World Medical Association Declaration of Helsinki, Seventh Revision, 2013).

Participants read the participant information sheet and gave informed consent prior to their participation in the experiment. Each participant kept a copy for their own records. Moreover, each participant received a total of £20 as reimbursement.

### Procedure

The experiment took place at the UCL Institute of Neurology, London, UK.

Prior to engaging in the experimental tasks, we asked participants to answer questions regarding age, gender, and sociodemographic information, as well as to complete the Generalized Anxiety Disorder scale version 7 (GAD-7) and the Patient Health Questionnaire version 9 (PHQ-9) (Kroenke et al., 2001; Löwe et al., 2008). These questionnaires were included to assess baseline mood and anxiety.

Each participant received one session of iTBS and one of sham stimulation separated by at least 3 days. The order of the sessions was randomized and counterbalanced in a single-blind within-subject design. Only participants were blinded to the condition, as the experimenter was responsible for implementing the protocol. The computer tasks were administered at three timepoints: prior to any sham/iTBS stimulation session (baseline), as well as once immediately after each sham/iTBS session. The testing session lasted for approximately 1 h. Participants were reimbursed £10 upon completion of each session.

### Measures

We used three emotion-processing tasks: the Emotional Categorization Word Task (ECAT) (adapted from the P1vital® Oxford Emotional Test Battery Tasks, n.d., and originally developed by Anderson, 1996), the Emotional Recognition Task (ERT) for faces, and the adapted Cambridge Gambling Task (CGT) (both from the EMOTICOM test battery developed by Bland et al., 2016). These tests assess three important aspects of affective cognition, specifically emotional word recall/categorization, facial emotion recognition, and decision-making, respectively. The total amount of time spent on the three tasks per session was approximately 20 min, and the order of presentation for the tasks was randomized, as well as the stimuli presented within the tasks.

#### ECAT word task

In this task, memory and accuracy for socially rewarding versus socially critical information were assessed. Each session comprised a set of 40 words indicative of personality traits (half of them likeable, half dislikeable); each set was different, and presented in random order for 500 ms. Participants indicated whether they would like or dislike to be described by others with each presented word by pressing a keyboard button. The ECAT was run on E-Run, an application within E-Prime software (Schneider et al., 2002). Once one session was completed, the experimenter gave the participant a free-recall test by asking them to write down as many words from the task as they could remember within 2 min, after which the experimenter calculated the number of positive/negative words recalled. The accuracy (positive/negative words correctly categorized as positive/negative) and reaction time for categorizing positive/negative words were also assessed. It has been suggested that self-relevant stimuli are processed automatically, and do not demand a high cognitive load, thus minimizing any potential practice effects (Bargh, 1982).

#### Emotion Recognition Task

The ERT for faces (Bland et al., 2016) measures the ability to identify emotions in facial expressions. The participant is required to indicate whether a face appearing on the screen is happy, sad, angry or fearful. The task was run on PsychoPy version 1.90.2 software (Peirce, 2007). The stimuli are morphed, averaged faces of real-life individuals expressing one of the four emotions, at 10 different intensities (where 1 is lowest, and 10 highest). The faces are presented for 250 ms, followed by four options (happy, sad, anger, fear) to choose from. Following each stimulus, a visual noise mask appears briefly to prevent afterimages. The 40 emotion-expressing stimuli are each repeated twice, totalling 80 faces presented to the participant. We calculated the number of happy, sad, anger and fear false alarms (i.e. other emotions incorrectly classified as happy, sad, angry and fearful) participants had for emotions in ambiguous faces (stimulus intensities 1–5), as well as the number of happy, sad, fear and anger hits (i.e. stimuli accurately identified as happy, sad, angry or fearful).

#### Cambridge Gambling Task

The adapted CGT (Bland et al., 2016) measures decision-making and risk-taking behaviour. Participants are presented with a pie chart – coloured orange and purple – depicting the probabilities of win and loss (50-50, 60-40, 70-30, 80-20 and 90-10 divisions), as well as sets of chips (5p, 10p and 20p stacks), from which participants must select two each time they place a bet on a pie division. In the win condition, participants bet two chips on a pie division, and either keep or double their bets, while in the loss condition they either keep or lose their bets. Thus, the conditions make a distinction between reward and punishment. There are a total of 15 trials per condition. The outcome measure is sensitivity to probability, assessed by the risk adjustment score (RA), which is calculated separately for the win and loss conditions using the following formula:

$$ \mathrm{RA}=\left(\left(2\ast \mathrm{bet}\ \mathrm{at}\ 90\%\right)+\left(1\ast \mathrm{bet}\ \mathrm{at}\ 80\%\right)+\left(0\ast \mathrm{bet}\ \mathrm{at}\ 70\%\right)-\left(1\ast \mathrm{bet}\ \mathrm{at}\ 60\%\right)-\left(2\ast \mathrm{bet}\ \mathrm{at}\ 50\%\right)\right)/\mathrm{Average}\ \mathrm{bet} $$

(Bland et al., 2016).

### Transcranial Magnetic Stimulation

For TMS, a biphasic Magstim Rapid^2^ stimulator with an eight-figure D70-mm alpha coil was used (Magstim Ltd, Whitland, Wales, UK). A 64-channel electroencephalogram (EEG) cap was set up according to the 10/20 system to determine the appropriate left DLPFC location, estimated to be at the F3 electrode (Fitzgerald et al., 2009). We followed these pre-established parameters in our study to ensure the quality of our findings.

Prior to administering iTBS/sham stimulation, the active motor threshold (AMT) was obtained for each participant by stimulating the left motor cortex while recording the response from the first dorsal interosseous (FDI) muscle of the right hand with Ag/AgCl surface electrodes arranged in a belly-tendon fashion. Raw signal, sampled at 5 kHz with a CED 1401 analog-to-digital laboratory interface (Cambridge Electronic Design Ltd.), was amplified and filtered (bandwidth 5 Hz–2 kHz) with a Digitimer D360 (Digitimer Ltd.). Data were stored on a laboratory computer for online visual display. AMT was defined as the lowest magnetic stimulator intensity able to evoke a motor-evoked potential of at least 200 μV in five out of ten trials (i.e. Bologna et al., 2016; Nardella et al., 2014; Rocchi et al., 2017).

After determining the appropriate intensity for stimulation, either sham or iTBS was administered in randomized, counterbalanced order to each participant. iTBS consisted of three-pulse bursts at 50 Hz, delivered in short trains lasting 2 s repeated every 10 s for 20 trains, for a total of 600 pulses. The stimulation intensity was set as 80% AMT, a value sufficiently strong to elicit an excitatory response (Bakker et al., 2015; Conte et al., 2014; Chung et al., 2017a; Chung et al., 2017b; Huang et al., 2005; Padberg & George, 2009). For the real iTBS, the coil was centred on F3 electrode at a 45° angle from the midline (Chung et al., 2017a; Fitzgerald et al., 2009), to elicit the strongest response (Thomson et al., 2013). For sham stimulation, the coil was oriented at 90° to the scalp so that the electromagnetic field was tangential to the head (Chung et al., 2017a).

### Statistical analysis

We performed multilevel mixed-effects linear regressions with individuals as a random intercept term to account for the two time-points for each outcome with Stata version 15 software (StataCorp, 2017). The independent variables were the administered stimulation (0 – sham; 1 – iTBS) and session order (1, 2, 3), with the dependent variables represented by the number of positive/negative words recalled, the reaction time and the accuracy to categorize positive/negative words in ECAT, the number of happy, sad, anger and fear false alarms for ambiguous faces and happy, sad, anger and fear hits participants made on the ERT, and the RA score for win/loss conditions in CGT, with each analysed in separate analyses.

The model was run before and after adjustment for confounder variables such as age, gender, education, mood and anxiety levels (PHQ-9 and the GAD-7 scores), which have been associated with both recall (Lewis et al., 2017) and emotion recognition (Harmer et al., 2003; Montagne et al., 2007). We also adjusted the model for the baseline measures (acquired prior to the first stimulation session) for each task.

We used alpha=0.05 (two-tailed) as our Type I error threshold. With 28 participants we had 80% power to detect an effect size (standardised mean difference) of d=0.55 between the stimulation conditions.

Moreover, we investigated if there are potential effects of order by running our models with session order as intercept term for each variable, while adjusting for potential confounding from stimulation type, age, gender, education and so on. We also looked at the unadjusted means and their corresponding standard deviations to get an overall idea of what performance looked like in each session.

## Results

Table 1 illustrates the participants’ demographic data. There were no missing values.

**Table 1:** Sample demographic distribution

Variable	Frequency	Percent
Gender		
Male	17	60.71
Female	11	39.29
Ethnicity		
White/White British	14	50
Black/Black British	2	7.14
Asian/Asian British	6	21.43
Mixed	3	10.71
Chinese	3	10.71
Marital status		
Married/living as married	3	10.71
Single	25	89.29
Education		
University	23	82.14
No university	5	17.86
Employment		
Student	22	78.57
Full-time work	4	14.29
Part-time work	2	7.14
Housing		
Tenant	23	82.14
Living with family/friends	3	10.71
Hostel/care home	1	3.57
Other	1	3.57
Financial situation		
Living comfortably	2	7.14
Doing alright	19	67.86
Just about getting by	7	25

*Overall sample population distribution by frequency and percentage

Scores on the PHQ-9 (mean 2.14; SD = 2.34; 95% CI: 1.24–3.05) and GAD-7 (mean 2; SD = 1.87; 95% CI: 1.28–2.72) indicated that participants had no significant mood or anxiety disruptions (scores of 5, 10 and 15 represent cut-off points for mild, moderate and severe depression and anxiety, respectively).

### ECAT Word Task

The mean and standard deviation for positive and negative words recalled, as well as the reaction time (RT) and accuracy (Acc) in categorizing positive and negative words, relative to the stimulation condition, are shown in Table 2. As seen in Table 3, iTBS increased the recall of positive words both before (mean difference = 1.29, 95% CI: 0.41–2.16, *P*-value = 0.004) and after adjustment (mean difference = 1.29, 95% CI: 0.53–2.04, *P*-value = 0.001). There was no evidence for an effect of iTBS on the recall of negative words (mean difference = -0.21, 95% CI: -1.06–0.63, *P-*value = 0.618 unadjusted; mean difference = -0.21, 95% CI: -0.97–0.54, *P-*value = 0.579 adjusted). Table 4 shows that iTBS did not have a significant effect on RT for positive (mean difference = 19.48, 95% CI: -69.88–108.83, *P-*value = 0.669 unadjusted; mean difference = 26.46, 95% CI: -49.70–102.62, *P-*value = 0.496 adjusted) or negative words (mean difference = -21.06, 95% CI: -79.31–37.19, *P-*value = 0.479 unadjusted; mean difference = -21.77, 95% CI: -72.68–29.15, *P-*value = 0.402 adjusted) as compared to sham stimulation, while Table 5 again shows no evidence for an effect of iTBS on accuracy (Acc) in categorizing positive (mean difference = -0.005, 95% CI: -0.04–0.02, *P-*value = 0.721 unadjusted; mean difference = -0.01, 95% CI: -0.04–0.02, *P-*value = 0.501 adjusted) or negative words (mean difference = 0.04, 95% CI: -0.004–0.08, *P-*value = 0.077 unadjusted; mean difference = 0.04, 95% CI: -0.002–0.07, *P-*value = 0.066 adjusted) when compared to sham stimulation.

**Table 2.** Means and standard deviation for recall of positive and negative words, reaction time (RT) to categorize positive and negative words, and accuracy or hits (Acc) to categorize positive and negative words relative to the sham and iTBS conditions, respectively (Emotional Categorization Word Task)

	Mean		SD	
	Sham	iTBS	Sham	iTBS
Positive words	5.07	6.36	2.09	2.47
Negative words	4.61	4.39	1.59	2.64
RT positive words	890.52	909.99	223.22	243.95
RT negative words	980.92	959.86	249.05	255.98
Acc positive words	0.93	0.92	0.08	0.07
Acc negative words	0.87	0.91	0.13	0.08

**Table 3.** Multilevel mixed-effects models unadjusted and adjusted, with positive and negative words recalled as dependent variables and stimulation (iTBS/sham) as independent variables, sham condition as reference value (Emotional Categorization Word Task)

	Unadjusted (N = 28)		*P*	Adjusted* (N = 28)		*P*
	Mean difference	95% CI		Mean difference	95% CI	
Positive words	1.29	0.41 to 2.16	0.004	1.29	0.53 to 2.04	0.001
Negative words	-0.21	-1.06 to 0.63	0.618	-0.21	-0.97 to 0.54	0.579

*Adjusted for age, gender, education, PHQ-9, GAD-7, session order, and baseline performance

**Table 4.** Multilevel mixed-effects models unadjusted and adjusted, with reaction time (RT) to categorize positive and negative words as dependent variables and stimulation (iTBS/sham) as independent variables, sham condition as reference value (Emotional Categorization Word Task)

	Unadjusted (N = 28)		*P*	Adjusted* (N = 28)		*P*
	Mean difference	95% CI		Mean difference	95% CI	
RT positive words	19.48	-69.88 to 108.83	0.669	26.46	-49.70 to 102.62	0.496
RT negative words	-21.06	-79.31 to 37.19	0.479	- 21.77	- 72.68 to 29.15	0.402

*Adjusted for age, gender, education, PHQ-9, GAD-7, session order, and baseline performance

**Table 5.** Multilevel mixed-effects models unadjusted and adjusted, with accuracy or hits (Acc) to categorize for positive and negative words as dependent variables and stimulation (iTBS/sham) as independent variables, sham condition as reference value (Emotional Categorization Word Task)

	Unadjusted (N = 28)		*P*	Adjusted* (N = 28)		*P*
	Mean difference	95% CI		Mean difference	95% CI	
Acc positive words	-0.005	-0.04 to 0.02	0.721	-0.01	-0.04 to 0.02	0.501
Acc negative words	0.04	-0.004 to 0.08	0.077	0.04	-0.002 to 0.07	0.066

*Adjusted for age, gender, education, PHQ-9, GAD-7, session order, and baseline performance

Tables 6, 7 and 8 show the effect of order of the sessions on each of the variables investigated, adjusted for potentially confounding variables (positive and negative words recalled in Table 6, RT positive words and RT negative words in Table 7, Acc positive words and Acc negative words in Table 8). There were effects of order present for recall of positive (mean difference = -0.97, 95% CI: -1.84–0.09, *P*-value = 0.029) and negative words (mean difference = 1.25, 95% CI: 0.45–2.06, *P*-value = 0.002), as well as concerning RT for categorizing both positive (mean difference = 110.03, 95% CI: 29.14–190.92, *P*-value = 0.008) and negative words (mean difference = 100.06, 95% CI: 47.24–152.88, *P*-value = 0.000). No effects of order were observed for accuracy (Acc) in categorizing positive (mean difference = -0.02, 95% CI: -0.05–0.02, *P*-value = 0.288) or negative words recalled (mean difference = -0.04, 95% CI: -0.08–0.002, *P*-value = 0.064).

**Table 6.** Adjusted* multilevel mixed-effects model with positive and negative words recalled as dependent variables and session order as independent variable, first session as reference value (Emotional Categorization Word Task)

	Mean difference	95% CI	*P*
Positive words	-0.97	-1.84 to -0.09	0.029
Negative words	1.25	0.45 to 2.06	0.002

*Adjusted for stimulation, age, gender, education, RT, Acc, PHQ-9, and GAD-7

**Table 7.** Adjusted* multilevel mixed-effects model with reaction time (RT) for categorizing positive and negative words as dependent variables and session order as independent variable, first session as reference value (Emotional Categorization Word Task)

	Mean difference	95% CI	*P*
RT positive words	110.03	29.14 to 190.92	0.008
RT negative words	100.06	47.24 to 152.88	0.000

*Adjusted for stimulation, age, gender, education, positive and negative words recalled, Acc, PHQ-9, and GAD-7

**Table 8.** Adjusted* multilevel mixed-effects model with accuracy (Acc) in categorizing positive and negative words as dependent variables and session order as independent variable, first session as reference value (Emotional Categorization Word Task)

	Mean difference	95% CI	*P*
Acc positive words	-0.02	-0.05 to 0.02	0.288
Acc negative words	-0.04	-0.08 to 0.002	0.064

*Adjusted for stimulation, age, gender, education, positive and negative words recalled, RT, PHQ-9, and GAD-7

**Fig. 1:**
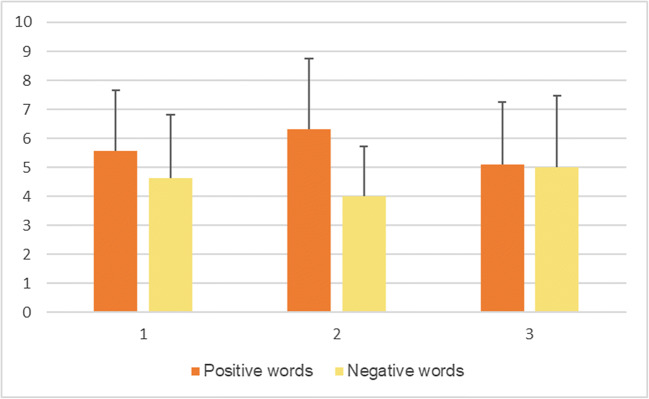
Unadjusted means for positive and negative words recalled per session, with error bars representing mean standard deviation

**Fig. 2:**
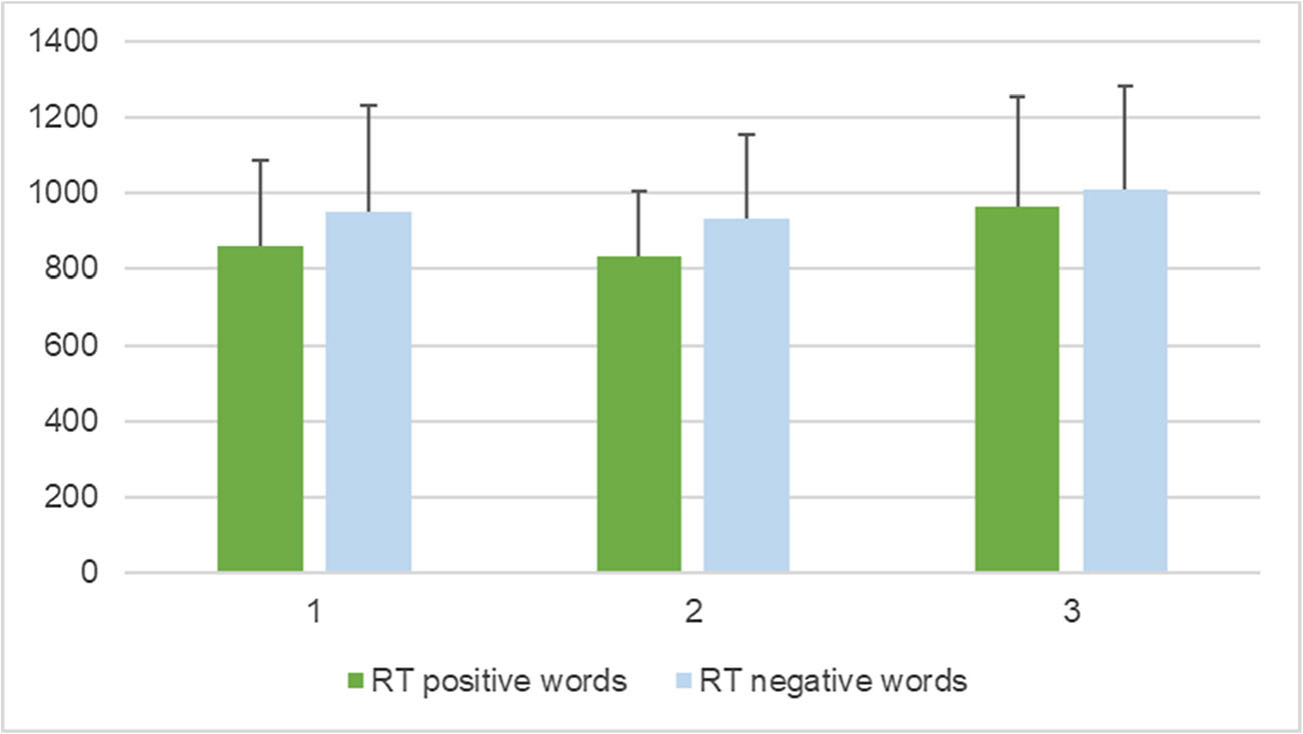
Unadjusted means for reaction time (RT) for categorizing positive and negative words per session, with error bars representing mean standard deviation

**Fig. 3:**
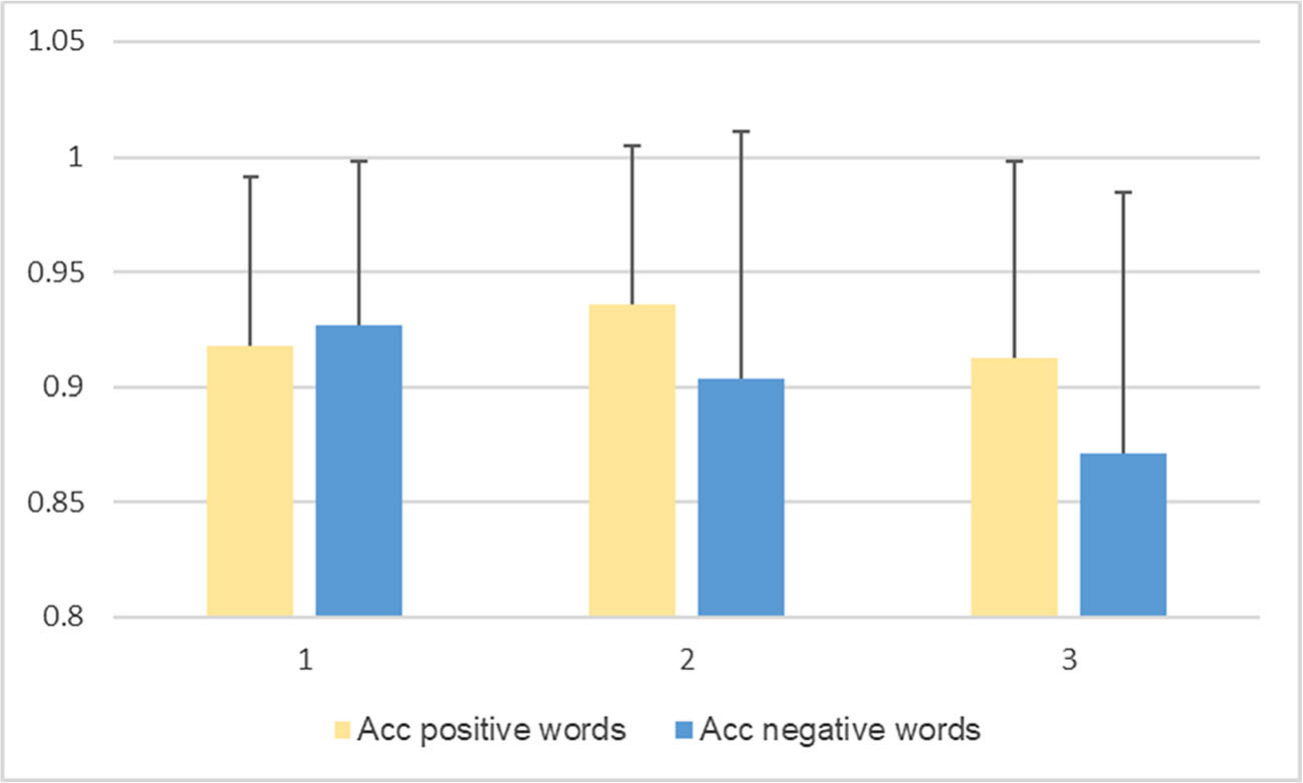
Unadjusted means for accuracy (Acc) for categorizing positive and negative words per session, with error bars representing mean standard deviation

Figures 1, 2, and 3 show the unadjusted means with corresponding standard deviations (SD) in each session for positive and negative words recalled (Fig. 1), reaction time (RT) for categorizing positive and negative words (Fig. 2), and accuracy (Acc) in categorizing positive and negative words (Fig. 3).

### Emotion Recognition Task

The mean and standard deviation for happy, sad, anger, and fear false alarms for ambiguous faces relative to the stimulation condition can be found in Table 9.

**Table 9.** Means and standard deviation for happy, sad, anger and fear false alarms relative to the sham and iTBS conditions, respectively (Emotion Recognition Task)

	Mean		SD	
	Sham	iTBS	Sham	iTBS
Happy false alarms	6.14	4.50	5.54	5.34
Sad false alarms	3.89	5.64	3.56	4.08
Anger false alarms	1.00	1.29	1.52	1.89
Fear false alarms	3.14	2.25	3.20	3.88

There were fewer happy false alarms in the iTBS than in the sham condition (mean difference = -1.64, 95% CI: -3.27 – -0.02, *P*-value = 0.047 before adjustment; mean difference = -1.64, 95% CI: -3.26 – -0.03, *P*-value = 0.046 after adjustment) and more sad false alarms in the iTBS condition than sham (mean difference = 1.75, 95% CI: 0.10–3.40, *P*-value = 0.037 before adjustment; mean difference = 1.75, 95% CI: 0.24–3.26, *P*-value = 0.023 after adjustment), while no difference between sham and iTBS conditions could be observed regarding fearful and angry false alarms (mean difference = 0.29, 95% CI: -0.72–1.29, *P*-value = 0.577 before adjustment for anger false alarms, and mean difference = 0.04, 95% CI: -0.82–0.89, *P*-value = 0.935 before adjustment for fear false alarms; mean difference = 0.32, 95% CI: -0.48–1.12, *P*-value = 0.435 after adjustment for anger false alarms, and mean difference = 0.05, 95% CI: -0.79–0.89, *P*-value = 0.904 after adjustment for fear false alarms) (Table 10).

**Table 10.** Multilevel mixed-effects models unadjusted and adjusted, with happy, sad, anger and fear false alarms as dependent variables and stimulation (iTBS/sham) as independent variables, sham condition as reference value (Emotion Recognition Task)

	Unadjusted (N = 28)		*P*	Adjusted for confounders* (N = 28)		*P*
	Mean difference	95% CI		Mean difference	95% CI	
Happy false alarms	-1.64	-3.27 to -0.02	0.047	-1.64	-3.26 to -0.03	0.046
Sad false alarms	1.75	0.10 to 3.40	0.037	1.75	0.24 to 3.26	0.023
Anger false alarms	0.29	-0.72 to 1.29	0.577	0.32	-0.48 to 1.12	0.435
Fear false alarms	0.04	-0.82 to 0.89	0.935	0.05	-0.79 to 0.89	0.904

*Adjusted for age, gender, education, PHQ-9, GAD-7, session order, and baseline performance

The means and standard deviations for happy, sad, anger, and fear hits for the sham and iTBS conditions can be found in Table 11. Regarding hits analyses, there were fewer happy hits in the iTBS than in the sham condition (mean difference = -0.05, 95% CI: -0.08 – -0.02, *P*-value = 0.001 before adjustment; mean difference = -0.04, 95% CI: -0.07 – -0.01, *P*-value = 0.008 after adjustment). There was no evidence for an effect of stimulation on sad hits (mean difference = 0.02, 95% CI: -0.02–0.06, *P*-value = 0.270 before adjustment; mean difference = -0.003, 95% CI: -0.04–0.03, *P*-value = 0.884 after adjustment), anger hits (mean difference = -0.02, 95% CI: -0.07–0.03, *P*-value = 0.478 before adjustment; mean difference = -0.02, 95% CI: -0.07–0.03, *P*-value = 0.373 after adjustment), or fear hits (mean difference = -0.01, 95% CI: -0.05–0.03, *P*-value = 0.579 before adjustment; mean difference = -0.01, 95% CI: -0.05–0.02, *P*-value = 0.532 after adjustment) (Table 12).

**Table 11.** Means and standard deviation for happy, sad, anger, and fear hits relative to the sham and iTBS conditions, respectively (Emotion Recognition Task)

	Mean		SD	
	Sham	iTBS	Sham	iTBS
Happy hits	0.94	0.89	0.09	0.12
Sad hits	0.78	0.80	0.16	0.14
Anger hits	0.67	0.65	0.16	0.17
Fear hits	0.83	0.82	0.14	0.14

**Table 12.** Multilevel mixed-effects models unadjusted and adjusted, with happy, sad, anger and fear hits as dependent variables and stimulations (iTBS/sham) as independent variables, sham condition as reference value (Emotion Recognition Task)

	Unadjusted (N = 28)		*P*	Adjusted for confounders* (N = 28)		*P*
	Mean difference	95% CI		Mean difference	95% CI	
Happy hits	-0.05	-0.08 to -0.02	0.001	-0.04	-0.07 to -0.01	0.008
Sad hits	0.02	-0.02 to 0.06	0.270	-0.003	-0.04 to 0.03	0.884
Anger hits	-0.02	-0.07 to 0.03	0.478	-0.02	-0.07 to 0.03	0.373
Fear hits	-0.01	-0.05 to 0.03	0.579	-0.01	-0.05 to 0.02	0.532

*Adjusted for age, gender, education, PHQ-9, GAD-7, session order, baseline performance, and happy and sad false alarms, respectively

**Fig. 4:**
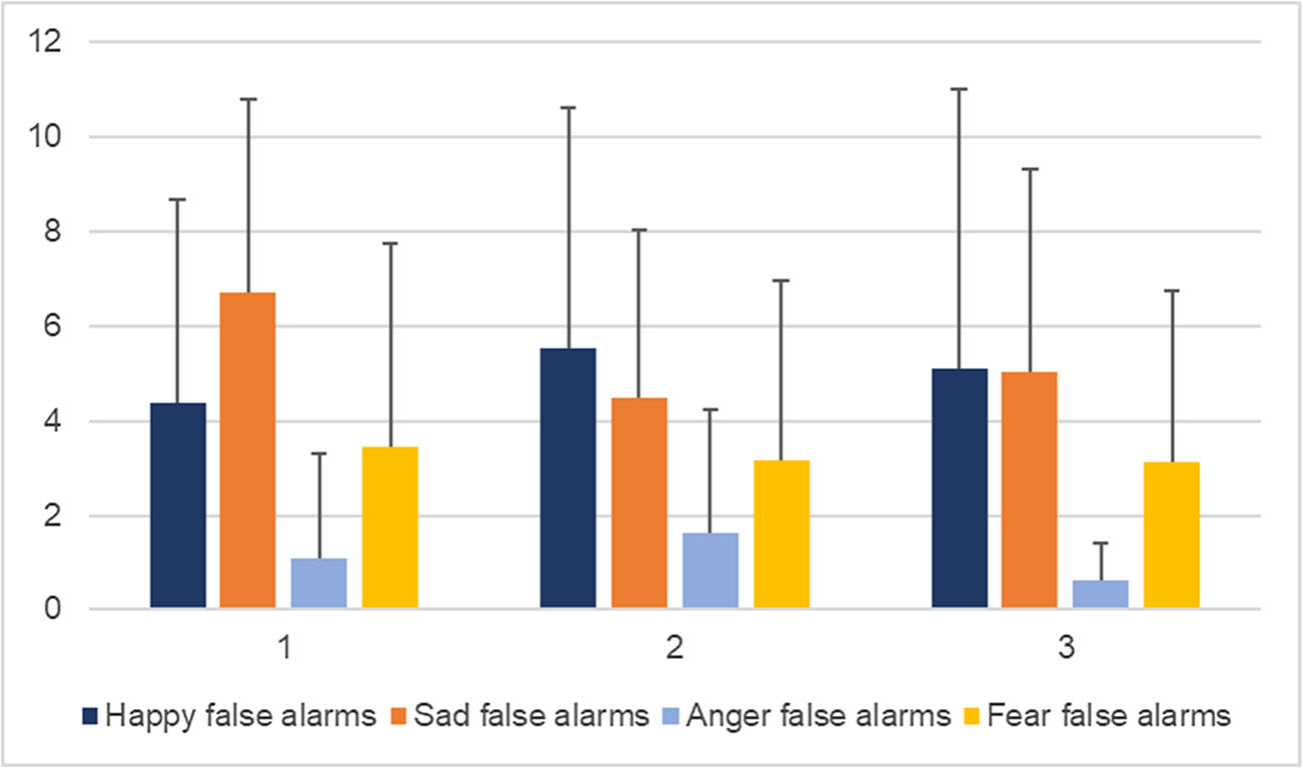
Unadjusted means for happy, sad, anger, and fear false alarms per session, with error bars representing mean standard deviation

**Fig. 5:**
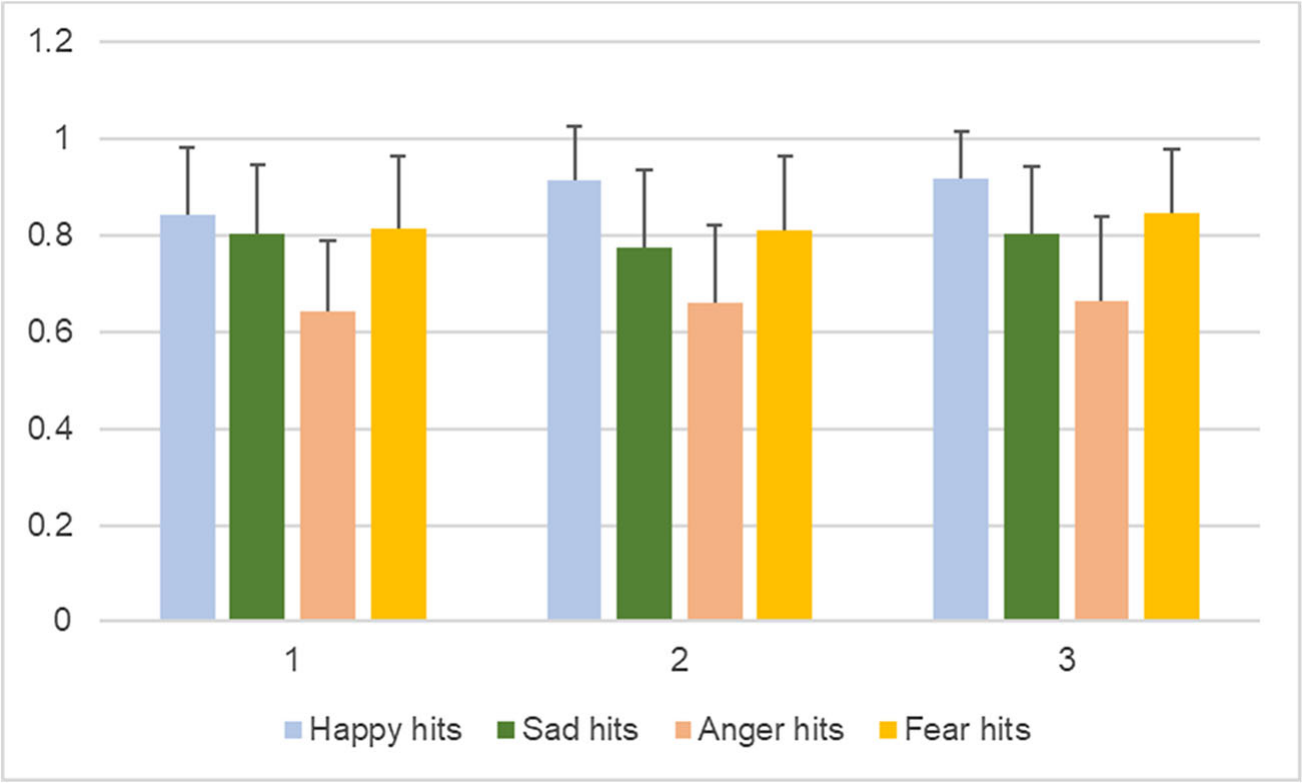
Unadjusted means for happy, sad, anger and fear hits per session, with error bars representing mean standard deviation

In Table 13, the sensitivity index (d’) is shown for each emotion (happy, sad, anger and fear, respectively) in each condition (sham and iTBS). Tables 14 and 15 show the effect of order of the sessions on each of the variables investigated (happy, sad, anger and fear false alarms, and happy, sad, anger and fear hits, respectively) irrespective of stimulation. Effects of order can only be observed for anger false alarms (mean difference = -1.00, 95% CI: -1.89 – -0.11, *P*-value = 0.028) and fear hits (mean difference = 0.04, 95% CI: 0.002–0.07, *P*-value = 0.037). Figures 4 and 5 depict the unadjusted means per session for happy, sad, anger, and fear false alarms (Fig. 4), and happy, sad, anger, and fear hits (Fig. 5) with their respective standard deviations expressed as error bars.

**Table 13.** Sensitivity index (d′) calculated based on the proportion of hits and of false alarms for happy, sad, anger and fear in the sham and iTBS conditions, respectively (Emotion Recognition Task)

	Sham	iTBS
Happy	2.84	2.63
Sad	2.25	2.07
Anger	2.49	2.27
Fear	2.51	2.47

**Table 14.** Adjusted* multilevel mixed-effects model with happy, sad, anger and fear false alarms for ambiguous faces as dependent variables and session (order) as independent variable, first session as reference value (Emotional Categorization Word Task)

	Mean difference	95% CI	*P*
Happy false alarms	-0.50	-2.19 to 1.19	0.564
Sad false alarms	0.16	-1.29 to 1.61	0.833
Anger false alarms	-1.00	-1.89 to -0.11	0.028
Fear false alarms	-0.28	-1.13 to 0.57	0.522

*Adjusted for stimulation, age, gender, education, hits, PHQ-9, and GAD-7

**Table 15.** Adjusted* multilevel mixed-effects model with happy, sad, anger and fear hits as dependent variables and session (order) as independent variable, first session as reference value (Emotional Categorization Word Task)

	Mean difference	95% CI	*P*
Happy hits	0.01	-0.03 to 0.04	0.687
Sad hits	0.02	-0.02 to 0.05	0.299
Anger hits	0.02	-0.03 to 0.07	0.531
Fear hits	0.04	0.002 to 0.07	0.037

*Adjusted for stimulation, age, gender, education, false alarms, PHQ-9, and GAD-7

#### Cambridge Gambling Task

Table 16 shows the mean and standard deviation for the win and loss conditions, relative to the sham and iTBS conditions. There was no evidence for an effect of stimulation on RA score (Table 17) in either the win (mean difference = 0.16, 95% CI: -0.35–0.68, *P*-value = 0.537 before adjustment; mean difference = 0.16, 95% CI: -0.35–0.68, *P*-value = 0.537 after adjustment) or the loss condition (mean difference = -0.21, 95% CI: -0.66–0.25, *P*-value = 0.372 before adjustment; mean difference = -0.21, 95% CI: -0.65–0.24, *P*-value = 0.359 after adjustment).

**Table 16.** Means and standard deviation for win and loss conditions relative to the sham and iTBS conditions, respectively (Cambridge Gambling Task)

	Mean		SD	
	Sham	iTBS	Sham	iTBS
Win	1.95	2.12	1.26	1.26
Loss	2.31	2.10	0.93	1.12

**Table 17.** Multilevel mixed-effects models unadjusted and adjusted, with win and loss conditions as dependent variables and stimulation (iTBS/sham) independent variables, sham condition as reference value (Cambridge Gambling Task)

	Unadjusted (N = 28)		*P*	Adjusted for confounders* (N = 28)		*P*
	Mean difference	95% CI		Mean difference	95% CI	
Win	0.16	-0.35 to 0.68	0.537	0.16	-0.35 to 0.68	0.537
Loss	-0.21	-0.66 to 0.25	0.372	-0.21	-0.65 to 0.24	0.359

*Adjusted for age, gender, education, PHQ-9, GAD-7, session order, and baseline performance

**Fig. 6:**
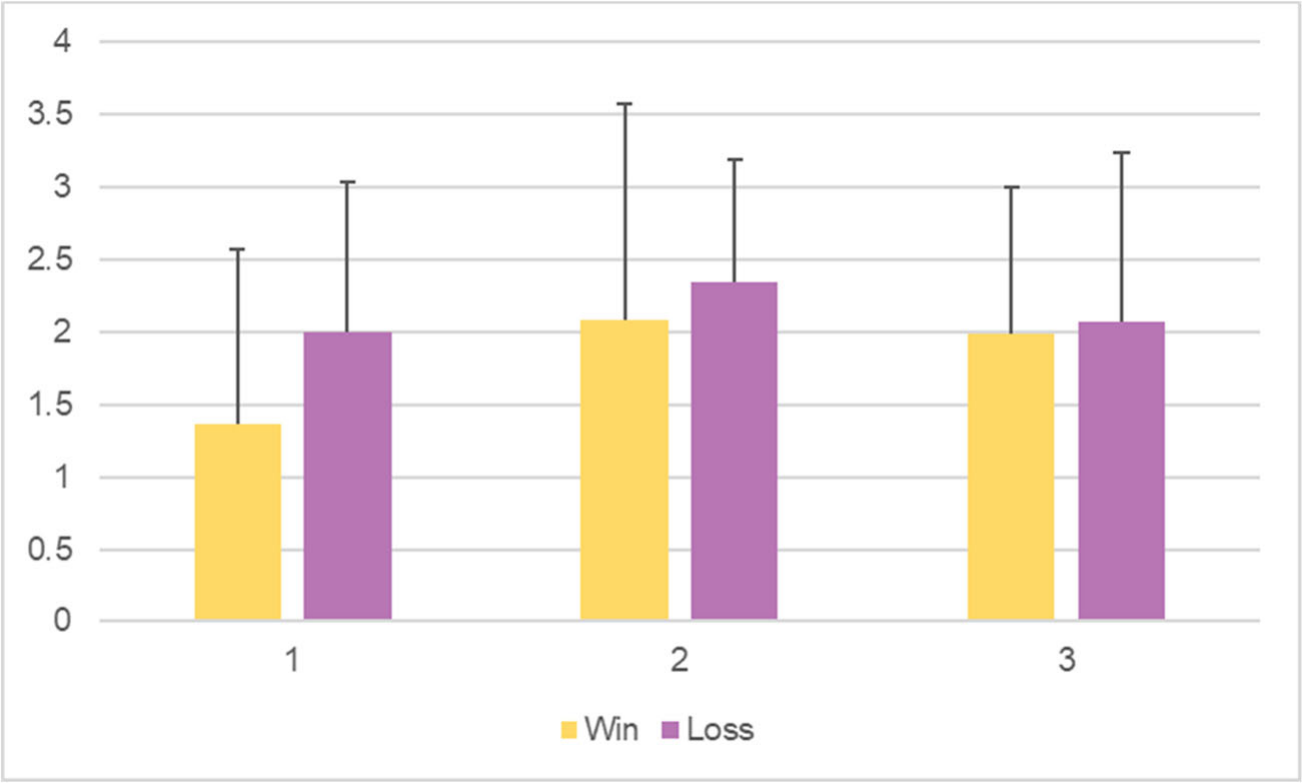
Unadjusted means for risk adjustment scores for win and loss conditions per session, with error bars representing mean standard deviation

Table 18 depicts the effect of order of the sessions on each of the variables investigated (win and loss) irrespective of stimulation. No effects of order of session can be observed for this task. Figure 6 shows the unadjusted means with standard deviation as error bars in each session for win and loss conditions.

**Table 18.** Adjusted* multilevel mixed-effects model with win and loss as dependent variables and session (order) as independent variable, first session as reference value (Cambridge Gambling Task)

	Mean difference	95% CI	*P*
Win	-0.09	-0.61 to 0.43	0.726
Loss	-0.27	-0.72 to 0.17	0.223

*Adjusted for stimulation, age, gender, education, PHQ-9, and GAD-7

## Discussion

iTBS over the left DLPFC influenced emotional word recall, with more positive words recalled after iTBS than after sham stimulation, in line with our prediction. However, it did not affect negative words recalled, reaction time (RT) or accuracy (Acc) in categorizing positive and negative words (ECAT). Counter to our hypothesis, there was evidence to support that iTBS led to an increased rate of miscategorization of ambiguous emotional faces as sad, relative to sham stimulation. Also contrary to our predictions, fewer happy false alarms were made for ambiguous faces, as well as fewer happy hits, following iTBS stimulation than sham. We found no evidence to suggest that iTBS over the left DLPFC has any effect on anger or fear false alarms, sad, anger or fear hits (ERT) or risk adjustment scores for the win and loss conditions (CGT).

Since iTBS appeared to change response bias in the ERT, we further analysed this using signal detection, and found that the probability of a hit as compared to a false alarm was above chance in both sham and iTBS conditions. However, as can be seen in Table 13, iTBS seems to slightly lower the d’ for each of the four emotions, meaning that iTBS over the left DLPFC appears to affects participants’ ability to accurately discriminate between emotional facial expressions, to some extent.

Overall, there was a partial confirmation of our hypothesis that iTBS over the left DLPFC would affect the emotion processing circuits that are also affected by monoaminergic antidepressants.

To our knowledge, this is the first study to investigate the effects of iTBS over the left DLPFC on three emotion processing domains – emotional word recall and categorization, emotion recognition in faces, and decision-making. Our results on the ECAT Word Task confirmed those of previous studies examining recall of socially and self-relevant words and its relation to depression (Harmer et al., 2009; Lewis et al., 2017). We used tasks validated within a depressive and a healthy population. We also followed appropriate methodology for determining accurate location and intensity of iTBS stimulation. Regarding the analysis, a multilevel mixed-effects model ensures precision of estimates of effect. The within-subject design allowed us to view effects at both the individual and group level, while adjusting for potential confounders. Considering the experimental nature of this study, as well as the randomization, we can be confident that we are measuring the effects of iTBS on cognition.

Some limitations of our study merit comment. First, as some participants were not native English speakers, they may have had difficulty processing the meaning or structure of some low-frequency words in ECAT. However, the within-subjects design prevents this type of bias affecting the results. Moreover, since we found an effect of iTBS on emotional word recall, the design would appear to be robust against such problems.

A second limitation is that, with the cross-over design, the tasks would be anticipated in the second exposure, though order effects were considered in the model, and adjusted for. Naturally, some practice effects did occur for RT and word recall. It appears that participants performed better in terms of RT in the second session for categorizing both positive and negative words, as compared to the first (baseline) and third. However, they appeared to perform the worst in the third session (trend can be observed in Fig. 2). They recalled the most positive words in the second session, as compared to the other two, while they recalled the least in the third session. For negative word recall, it is reversed – the most recalled were in the third session, while the least were in the second one (trend can be observed in Fig. 1). Session order effects can be seen for anger false alarms (trend observed in Fig. 4) and fear hits (Fig. 5), but not the other variables, when controlled for stimulation. This suggests that, as time progressed, participants became better at identifying fearful faces, and less inclined to have anger false alarms. Bentin and Moscovitch proposed that practice effects are affected by “the type of stimulus, its pre-experimental history, the level to which it is processed, and the lag between the initial presentation and the test” (1988). Indeed, the most interesting differences are seen between the baseline session and the second session of tasks, which took place on the same day, while the third session, as the iTBS stimulation protocol required, took place after at least 3 days. Reeve and Lam (2005) observed measurement invariance across repeated task administration, which is a potential explanation for these effects of order observed only for some variables, and not for others. Potential unexplained factors, unaccounted for by the experimenters, could have also played a part in these order effects.

A third potential limitation is possible observer bias introduced by the single-blind nature of the design; we made efforts to limit this possibility using objective cognitive tests as outcome measures. However, this represents an inherent limitation of rTMS studies: the impossibility of double blinding, as applying the stimulation requires knowledge of the protocols. Fourth, the sample size (N = 28) was adequate, but relatively small to detect subtle differences, which is evident in the broad confidence intervals. As these intervals are quite wide, our estimates have lower precision, but the within-subject design affords greater statistical power than would be achieved with between-subjects design with the same sample size.

We recorded PHQ-9 and GAD-7 scores before any type of stimulation or task were administered, thus adjusting for baseline psychopathology. The range of scores in our healthy volunteer sample was narrow and in the normal range, although future work should examine whether such scores change after iTBS stimulation. Hence, a direct relationship between iTBS, emotion processing tasks, and depression cannot be established. Furthermore, direct inferences at the neurophysiological level cannot be made on the action of iTBS in the absence of neuroimaging or related methods. Further research should try to address these issues by using a combination of neuroimaging and behavioural methods in a clinical sample.

## Conclusions and implications

iTBS over the left DLPFC influenced the recall of socially and emotionally rewarding words, as participants recalled more positive words after iTBS than after sham, with no difference between conditions for negative words. This was consistent with our hypothesis and with previous research involving a clinical population (Lewis et al., 2017), as well as that involving antidepressant administration (Harmer et al., 2009), indicating that iTBS over the left DLPFC might have a similar mechanism of action to that of antidepressants, by increasing positive affective bias in emotional word recall. No difference could be seen between the stimulation conditions for reaction time and accuracy to categorize positive and negative words, meaning that iTBS only has an effect with regard to memory (recall) for positive words, but not for categorization. This finding, specifically with respect to memory for positive words, is supported by Balconi and Cobelli’s research (2015), where they found that left lateralized rTMS induces a performance improvement in positive emotional words. Additionally, left DLPFC activation via rTMS is believed to enhance memory retrieval of emotional information (Balconi & Ferrari, 2012b; Balconi & Cobelli, 2014), which further confirms our findings regarding the ECAT recall of positive words post-iTBS. The relevance of these findings lies in the psychopathological realm, as memory for positive stimuli is blunted by psychiatric disturbances, such as depression and anxiety. A good example that looks particularly at stimulation effects on memory for anxious people is a study by Balconi and Ferrari (2013), who investigated the effects of rTMS over the left DLPFC on memory in high and low anxiety participants, and found that high anxiety participants benefitted the most from stimulation, as repeated stimulation improved their performance with respect to both memory encoding and retrieval. Skrdlantová et al. also found that memory for words is quite localized to the left prefrontal cortex, and that rTMS can tap into that network (2005).

With respect to emotion recognition in faces, the findings did not support our hypothesis. Overall, participants showed reduced happy hits and happy false alarms, and increased sad false alarms for ambiguous faces in the iTBS condition. This finding was inconsistent with previous studies that found antidepressants to increase recognition of positive faces (Harmer et al., 2009), as well as other brain stimulation studies which showed an improved affective processing of positive stimuli (Baeken et al., 2011; Nitsche et al., 2012; Ozcan, Gica, & Gulec, 2020). However, Balconi and Canavesio (2016) found a similar reduced performance regarding happy face recognition after TMS stimulation on the left DLPFC. There were no effects found for anger and fear hits, nor for anger and fear false alarms after iTBS as compared to sham stimulation. This finding also contrasts with previous literature, as stimulation of the DLPFC has been shown to have an effect on processing of angry faces. Studies in which the left DLPFC was stimulated have seen diminished engagement with angry faces (De Raedt et al.*,* 2010; Mondino, Thiffault, & Fecteau, 2015; Moulier et al.*,* 2016) associated with increased activity in other areas of the brain, such as the right DLPFC, the left orbitofrontal cortex, and the dorsal anterior cingulate cortex. For fearful faces, however, the literature suggests that there is a general amplified response after inhibitory rTMS to the right DLPFC (Zwanzger et al., 2014), not the left, as the right DLPFC appears to modulate facial fear-specific reactions (Ran et al., 2016). The dorsomedial PFC also seems to be involved in this facial processing circuit, as rTMS seems to worsen performance for angry and fearful faces (Balconi and Bortolotti, 2012). It is possible, then, that iTBS to the left DLPFC disrupts the circuitry involved in processing happy and sad faces, while it does not particularly affect anger and fearful face perception. A problem for interpretation is the complexity regarding the neural correlates of emotional recognition in faces. Emotion recognition of faces appears to be bilaterally distributed within the brain (Haxby et al., 2000), with the amygdala becoming reactive upon viewing emotional faces, while prefrontal regions are only involved when actively identifying specific emotions (Adolphs, 2002). Moreover, different neural systems are involved in the perception of different emotional expressions. For instance, a study found two distinct neural substrates in response to sad and angry faces (Blair et al., 1999), and another pinpointed distinct neuronal response to happy and fearful emotion-expressing faces (Kawasaki et al., 2001). Another study showed that TMS to the medial-frontal cortex impairs the processing of angry faces (Harmer et al., 2001). As such, it is possible that iTBS over the left DLPFC might be tapping into different neural systems, which may not yield a simple increase or decrease in emotion recognition bias. Recent results from our work have also suggested that the relationship between depression and facial emotion recognition is somewhat more complex than some of the previous research has suggested (i.e. Bone et al., 2019). Further work exploring other neuromodulatory targets might clarify the picture; however, we are still far from understanding the complexity involved in the processing of emotional facial stimuli, which seems to be connected throughout the brain, as opposed to being localized, as previously believed. For instance, Skrdlantová et al. (2005) found that left prefrontal rTMS has a clear influence on words, but not faces, suggesting that facial emotion processing is not localized to the left prefrontal regions, but spread across multiple areas of the brain. Consequently, accessing these connections would require further research with fine-tuned methodology to create a better understanding of facial emotion processing and encoding circuitry.

Finally, we did not find any evidence that iTBS influenced performance on the CGT. Recent unpublished work in a large birth cohort did not find a relationship between CGT performance and depressive symptoms in adolescence. Perhaps different brain mechanisms are involved in performance on the CGT. Tulviste and Bachmann (2018) found that rTMS to the right DLPFC decreases risk-aversion, so it is possible that the left DLPFC is not actively involved in this pathway. Yang, Khalifa, and Völlm (2018a) found no significant changes regarding impulsivity following excitatory rTMS applied to the right inferior frontal gyrus. Later that year, Yang et al. (2018b) published a paper on a similar experiment as the present one, where they investigated iTBS over the left DLPFC for impulsivity in healthy adult males, and, like us, found no significant effect of iTBS on impulsivity as captured by the CGT. These findings could indicate a distinct neural pathway for emotional decision-making when stimulating the PFC. In contrast, Cho et al. (2010) compared cTBS, iTBS, and sham over the right DLPFC, to find that iTBS had no effect, and only cTBS modulated impulsivity. Then, it could be argued that stimulation protocol is perhaps as relevant as stimulated area in eliciting different responses with respect to decision-making.

To conclude, iTBS is a time-efficient method of stimulating the brain, with great potential for timely treatment and alleviation for symptoms of depression and other psychiatric disorders. We have provided some evidence to support the hypothesis that iTBS affects circuits involved in emotion processing and these are the same processes that are affected by antidepressant medication. Our findings do not support the idea that emotion facial recognition can be influenced significantly by means of iTBS to the left DLPFC, suggesting that emotion recognition for faces is not localized, and that the literature so far is inconsistent on the neural pathways behind it. Further studies performed in clinically depressed populations could bridge existing knowledge and aid in understanding the mechanism of action for iTBS on depression, while neuroimaging and physiological methods could help fill the gaps regarding brain pathways and mechanisms, to ultimately inform proper protocols for brain stimulation use. It is essential that we further investigate such topics, not only to improve current research tools, but to uncover the underlying neural circuitries, so that we can develop better treatment options and build upon existing knowledge, in understanding the DLPFC-rTMS relationship.

## References

[CR1] Adolphs R (2002). Neural systems for recognizing emotion. Current Opinion in Neurobiology.

[CR2] Amick HR, Gartlehner G, Gaynes BN, Forneris C, Asher GN, Morgan LC (2015). Comparative benefits and harms of second generation antidepressants and cognitive behavioral therapies in initial treatment of major depressive disorder: systematic review and meta-analysis. BMJ.

[CR3] Anderson NH (1996). Likableness ratings of 555 personality-trait words. Journal of Personality and Social Psychology.

[CR4] Baeken C, De Raedt R (2011). Neurobiological mechanisms of repetitive transcranial magnetic stimulation on the underlying neuro circuitry in unipolar depression. Dialogues in Clinical Neuroscience.

[CR5] Baeken C, Van Schuerbeek P, De Raedt R, De Mey J, Vanderhasselt MA, Bossuyt A (2011). The effect of one left-sided dorsolateral prefrontal sham-controlled HF-rTMS session on approach and withdrawal related emotional neuronal processes. Clinical Neurophysiology.

[CR6] Bakker N, Shahab S, Giacobbe P, Blumberger DM, Daskalakis ZJ, Kennedy SH (2015). rTMS of the dorsomedial prefrontal cortex for major depression: safety, tolerability, effectiveness, and outcome predictors for 10 Hz versus intermittent theta-burst stimulation. Brain Stimulation.

[CR7] Balconi M, Bortolotti A (2012). Emotional face recognition, empathic trait (BEES), and cortical contribution in response to positive and negative cues. The effect of rTMS on dorsal medial prefrontal cortex. Cognitive Neurodynamics.

[CR8] Balconi M, Canavesio Y (2016). Empathy, Approach Attitude, and rTMs on Left DLPFC Affect Emotional Face Recognition and Facial Feedback (EMG). Journal of Psychophysiology.

[CR9] Balconi M, Cobelli C (2014). Motivational mechanisms (BAS) and prefrontal cortical activation contribute to recognition memory for emotional words. rTMS effect on performance and EEG (alpha band) measures. Brain and Language.

[CR10] Balconi M, Cobelli C (2015). rTMS on left prefrontal cortex contributes to memories for positive emotional cues: A comparison between pictures and words. Neuroscience.

[CR11] Balconi M, Ferrari C (2012). rTMS stimulation on left DLPFC increases the correct recognition of memories for emotional target and distractor words. Cognitive, Affective, & Behavioral Neuroscience.

[CR12] Balconi M, Ferrari C (2012). Emotional memory retrieval. rTMS stimulation on left DLPFC increases the positive memories. Brain Imaging and Behavior.

[CR13] Balconi M, Ferrari C (2013). Repeated transcranial magnetic stimulation on dorsolateral prefrontal cortex improves performance in emotional memory retrieval as a function of level of anxiety and stimulus valence. Psychiatry and Clinical Neurosciences.

[CR14] Bargh J (1982). Attention and automaticity in the processing of self-relevant information. Journal of Personality and Social Psychology.

[CR15] Barth J, Munder T, Gerger H, Nüesch E, Trelle S, Znoj H (2013). Comparative efficacy of seven psychotherapeutic interventions for patients with depression: A network meta-analysis. PLoS Medicine.

[CR16] Bentin S, Moscovitch M (1988). The time course of repetition effects for words and unfamiliar faces. Journal of Experimental Psychology: General.

[CR17] Blair RJR, Morris JS, Frith CD, Perrett DI, Dolan RJ (1999). Dissociable neural responses to facial expressions of sadness and anger. Brain.

[CR18] Bland, A.R., Roiser, J.P., Mehta, M.A., Schei, T., Boland, H., Campbell-Meiklejohn, D.K., *et al*. (2016). EMOTICOM: A Neuropsychological Test Battery to Evaluate Emotion, Motivation, Impulsivity, and Social Cognition. Frontiers in Behavioral Neuroscience, 10. 10.3389/fnbeh.2016.00025 PMC4764711 26941628

[CR19] Blumberger DM, Vila-Rodriguez F, Thorpe KE, Feffer K, Noda Y, Giacobbe P (2018). Effectiveness of theta burst versus high-frequency repetitive transcranial magnetic stimulation in patients with depression (THREE-D): a randomised non-inferiority trial. The Lancet.

[CR20] Bologna M, Paparella G, Fabbrini A, Leodori G, Rocchi L (2016). Effects of cerebellar theta-burst stimulation on arm and neck movement kinematics in patients with focal dystonia. Clinical Neurophysiology.

[CR21] Bone JK, Lewis G, Button KS, Duffy L, Harmer CJ, Munafò MR (2019). Variation in recognition of happy and sad facial expressions and self-reported depressive symptom severity: A prospective cohort study. Journal of Affective Disorders.

[CR22] Brunoni AR, Zanao TA, Vanderhasselt M-A, Valiengo L, de Oliveira JF, Boggio PS (2014). Enhancement of Affective Processing Induced by Bifrontal Transcranial Direct Current Stimulation in Patients with Major Depression. Neuromodulation: Technology at the Neural Interface.

[CR23] Cacioppo, J.T., Crites, Jr. S.L., Berntson, G.G., & Coles, M.G. (1993). If attitudes affect how stimuli are processed, should they not affect the event-related brain potential? Psychological Science, 4(2), 108–112.

[CR24] Cho SS, Ko JH, Pellecchia G, Van Eimeren T, Cilia R, Strafella AP (2010). Continuous theta burst stimulation of right dorsolateral prefrontal cortex induces changes in impulsivity level. Brain Stimulation.

[CR25] Choi, K. M., Scott, D. T., & Lim, S.-L. (2016). The modulating effects of brain stimulation on emotion regulation and decision-making. Neuropsychiatric Electrophysiology, 2(1).

[CR26] Chung SW, Lewis BP, Rogasch NC, Saeki T, Thomson RH, Hoy KE (2017). Demonstration of short-term plasticity in the dorsolateral prefrontal cortex with theta burst stimulation: A TMS-EEG study. Clinical Neurophysiology.

[CR27] Chung SW, Rogasch NC, Hoy KE, Sullivan CM, Cash RFH, Fitzgerald PB (2017). Impact of different intensities of intermittent theta burst stimulation on the cortical properties during TMS-EEG and working memory performance. Human Brain Mapping.

[CR28] Cipriani A, Furukawa TA, Salanti G, Chaimani A, Atkinson LZ, Ogawa Y (2018). Comparative efficacy and acceptability of 21 antidepressant drugs for the acute treatment of adults with major depressive disorder: a systematic review and network meta-analysis. The Lancet.

[CR29] Conte A, Rocchi L, Ferrazzano G, Leodori G, Bologna M, Li Voti P (2014). Primary somatosensory cortical plasticity and tactile temporal discrimination in focal hand dystonia. Clinical Neurophysiology.

[CR30] Cramer SC, Sur M, Dobkin BH, O’Brien C, Sanger TD, Trojanowski JQ (2011). Harnessing neuroplasticity for clinical applications. Brain.

[CR31] Cuijpers P, Huibers MJH, Furukawa TA (2017). The need for research on treatments of chronic depression. JAMA Psychiatry.

[CR32] De Raedt R, Leyman L, Baeken C, Van Schuerbeek P, Luypaert R, Vanderhasselt (2010). Neurocognitive effects of HF-rTMS over the dorsolateral prefrontal cortex on the attentional processing of emotional information in healthy women: An event-related fMRI study. Biological Psychology.

[CR33] Dolcos F, LaBar KS, Cabeza R (2004). Dissociable effects of arousal and valence on prefrontal activity indexing emotional evaluation and subsequent memory: an event-related fMRI study. NeuroImage.

[CR34] Eshel N, Huemer J, McTeague L, Wong M, Yee A, Patenaude B (2017). 639. Effect of rTMS on resting-state functional connectivity in patients with major depression. Biological Psychiatry.

[CR35] Fales CL, Barch DM, Rundle MM, Mintun MA, Snyder AZ, Cohen JD (2008). Altered Emotional Interference Processing in Affective and Cognitive-Control Brain Circuitry in Major Depression. Biological Psychiatry.

[CR36] Fitzgerald PB, Hoy KE, Anderson RJ, Daskalakis ZJ (2016). A study of the pattern of response to rTMS treatment in depression. Depression and Anxiety.

[CR37] Fitzgerald PB, Maller JJ, Hoy KE, Thomson R, Daskalakis ZJ (2009). Exploring the optimal site for the localization of dorsolateral prefrontal cortex in brain stimulation experiments. Brain Stimulation.

[CR38] George MS (1998). Why would you ever want to?: Toward understanding the antidepressant effect of prefrontal rTMS. Human Psychopharmacology.

[CR39] George MS, Post RM (2011). Daily left prefrontal repetitive transcranial magnetic stimulation for acute treatment of medication-resistant depression. American Journal of Psychiatry.

[CR40] Grimm S, Beck J, Schuepbach D, Hell D, Boesiger P, Bermpohl F (2008). Imbalance between left and right dorsolateral prefrontal cortex in major depression is linked to negative emotional judgment: An fMRI study in severe Major Depressive Disorder. Biological Psychiatry.

[CR41] Groenewegen HJ, Uylings HBM (2000). The prefrontal cortex and the integration of sensory, limbic and autonomic information. Progress in Brain Research.

[CR42] Guo, Z., Jiang, Z., Jiang, B., McClure, M. A., & Mu, Q. (2019). High-Frequency Repetitive Transcranial Magnetic Stimulation Could Improve Impaired Working Memory Induced by Sleep Deprivation. Neural Plasticity, 2019, 1–11. 10.1155/2019/7030286 PMC6930796 31915432

[CR43] Harmer CJ, Bhagwagar Z, Perrett DI, Völlm BA, Cowen PJ, Goodwin GM (2003). Acute SSRI administration affects the processing of social cues in healthy volunteers. Neuropsychopharmacology.

[CR44] Harmer CJ, O’Sullivan U, Favaron E, Massey-Chase R, Ayres R, Reinecke A (2009). Effect of acute antidepressant administration on negative affective bias in depressed patients. American Journal of Psychiatry.

[CR45] Harmer CJ, Thilo KV, Rothwell JC, Goodwin GM (2001). Transcranial magnetic stimulation of medial–frontal cortex impairs the processing of angry facial expressions. Nature Neuroscience.

[CR46] Haxby JV, Hoffman EA, Gobbini MI (2000). The distributed human neural system for face perception. Trends in Cognitive Sciences.

[CR47] Huang YZ, Edwards MJ, Rounis E, Bhatia KP, Rothwell JC (2005). Theta burst stimulation of the human motor cortex. Neuron.

[CR48] Ironside, M., O’Shea, J., Cowen, P. J., & Harmer, C. J. (2016). Frontal Cortex Stimulation Reduces Vigilance to Threat: Implications for the Treatment of Depression and Anxiety. Biological Psychiatry, 79(10), 823–830. 10.1016/j.biopsych.2015.06.012 26210058

[CR49] Kawasaki H, Adolphs R, Kaufman O, Damasio H, Damasio AR, Granner M (2001). Single-unit responses to emotional visual stimuli recorded in human ventral prefrontal cortex. Nature Neuroscience.

[CR50] Kito S, Hasegawa T, Takamiya A, Noda T, Nakagome K, Higuchi T (2017). Transcranial magnetic stimulation modulates resting EEG functional connectivity between the left dorsolateral prefrontal cortex and limbic regions in medicated patients with treatment-resistant depression. The Journal of Neuropsychiatry and Clinical Neurosciences.

[CR51] Klomjai W, Katz R, Lackmy-Vallée A (2015). Basic principles of transcranial magnetic stimulation (TMS) and repetitive TMS (rTMS). Annals of Physical and Rehabilitation Medicine.

[CR52] Koenigs M, Grafman J (2009). The functional neuroanatomy of depression: Distinct roles for ventromedial and dorsolateral prefrontal cortex. Behavioural Brain Research.

[CR53] Kroenke K, Spitzer RL, Williams JBW (2001). The PHQ-9. Journal of General Internal Medicine.

[CR54] Lewis G, Kounali DZ, Button KS, Duffy L, Wiles NJ, Munafò MR (2017). Variation in the recall of socially rewarding information and depressive symptom severity: a prospective cohort study. Acta Psychiatrica Scandinavica.

[CR55] Li Y, Wang L, Jia M, Guo J, Wang H, Wang M (2017). The effects of high-frequency rTMS over the left DLPFC on cognitive control in young healthy participants. PLOS ONE.

[CR56] Löwe B, Decker O, Müller S, Brähler E, Schellberg D, Herzog W (2008). Validation and standardization of the Generalized Anxiety Disorder Screener (GAD-7) in the general population. Medical Care.

[CR57] Mondino, M., Thiffault, F., & Fecteau, S. (2015). Does non-invasive brain stimulation applied over the dorsolateral prefrontal cortex non-specifically influence mood and emotion processing in healthy individuals? Frontiers in Cellular Neuroscience, 9. 10.3389/fncel.2015.00399 PMC4604238 26528131

[CR58] Montagne B, Kessels RPC, De Haan EHF, Perrett DI (2007). The Emotion Recognition Task: A paradigm to measure the perception of facial emotional expressions at different intensities. Perceptual and Motor Skills.

[CR59] Moulier V, Gaudeau-Bosma C, Isaac C, Allard A-C, Bouaziz N, Sidhoumi D (2016). Effect of repetitive transcranial magnetic stimulation on mood in healthy subjects. Socioaffective Neuroscience & Psychology.

[CR60] Nardella A, Rocchi L, Conte A, Bologna M, Suppa A, Berardelli A (2014). Inferior Parietal Lobule Encodes Visual Temporal Resolution Processes Contributing to the Critical Flicker Frequency Threshold in Humans. PLoS ONE.

[CR61] Nitsche, M. A., Koschack, J., Pohlers, H., Hullemann, S., Paulus, W., & Happe, S. (2012). Effects of Frontal Transcranial Direct Current Stimulation on Emotional State and Processing in Healthy Humans. Frontiers in Psychiatry, 3. 10.3389/fpsyt.2012.00058 PMC3377009 22723786

[CR62] Ozcan S, Gica S, Gulec H (2020). Suicidal behavior in treatment resistant major depressive disorder patients treated with transmagnetic stimulation (TMS) and its relationship with cognitive functions. Psychiatry Research.

[CR63] P1vital® Oxford Emotional Test Battery Tasks. (n.d). Available at: http://www.p1vital.com/Oxford%20Emotional%20Test%20Battery/index.html. Accessed Sep 2018.

[CR64] Padberg F, George MS (2009). Repetitive transcranial magnetic stimulation of the prefrontal cortex in depression. Experimental Neurology.

[CR65] Pascual-Leone A, Rubio B, Pallardó F, Catalá MD (1996). Rapid-rate transcranial magnetic stimulation of left dorsolateral prefrontal cortex in drug-resistant depression. The Lancet.

[CR66] Peirce JW (2007). PsychoPy - Psychophysics software in Python. Journal of Neuroscientific Methods.

[CR67] Philiastides MG, Auksztulewicz R, Heekeren HR, Blankenburg F (2011). Causal Role of Dorsolateral Prefrontal Cortex in Human Perceptual Decision Making. Current Biology.

[CR68] Ran, G., Chen, X., Zhang, Q., Ma, Y., & Zhang, X. (2016). Attention Modulates Neural Responses to Unpredictable Emotional Faces in Dorsolateral Prefrontal Cortex. Frontiers in Human Neuroscience, 10. 10.3389/fnhum.2016.00332 PMC4923193 27445769

[CR69] Reeve CL, Lam H (2005). The psychometric paradox of practice effects due to retesting: Measurement invariance and stable ability estimates in the face of observed score changes. Intelligence.

[CR70] Ridding MC, Rothwell JC (2007). Is there a future for therapeutic use of transcranial magnetic stimulation?. Nature Reviews Neuroscience.

[CR71] Rocchi L, Erro R, Antelmi E, Berardelli A, Tinazzi M, Liguori R (2017). High frequency somatosensory stimulation increases sensori-motor inhibition and leads to perceptual improvement in healthy subjects. Clinical Neurophysiology.

[CR72] Rock PL, Roiser JP, Riedel WJ, Blackwell AD (2014). Cognitive impairment in depression: a systematic review and meta-analysis. Psychological Medicine.

[CR73] Roiser JP, Elliott R, Sahakian BJ (2011). Cognitive Mechanisms of Treatment in Depression. Neuropsychopharmacology.

[CR74] Roiser, J. P., & Sahakian, B. J. (2016). Information Processing in Mood Disorders. Oxford Handbooks Online.

[CR75] Schneider W, Eschman A, Zuccolotto A (2002). E-Prime Reference Guide.

[CR76] Schutter DJ, van Honk J (2005). A framework for targeting alternative brain regions with repetitive transcranial magnetic stimulation in the treatment of depression. J. Psychiatry Neurosci..

[CR77] Silvanto J, Pascual-Leone A (2008). State-dependency of transcranial magnetic stimulation. Brain Topography.

[CR78] Skrdlantová L, Horácek J, Dockery C, Lukavský J, Kopeček M, Preiss M (2005). The influence of low-frequency left prefrontal repetitive transcranial magnetic stimulation on memory for words but not for faces. Physiological Research.

[CR79] StataCorp (2017). Stata Statistical Software: Release.

[CR80] Suppa A, Huang YZ, Funke K, Ridding MC, Cheeran B, Di Lazzaro V (2016). Ten years of theta burst stimulation in humans: Established knowledge, unknowns and prospects. Brain Stimulation.

[CR81] Thomson RH, Cleve TJ, Bailey NW, Rogasch NC, Maller JJ, Daskalakis ZJ (2013). Blood oxygenation changes modulated by coil orientation during prefrontal transcranial magnetic stimulation. Brain Stimulation.

[CR82] Tulviste J, Bachmann T (2018). Diminished Risk-Aversion After Right DLPFC Stimulation: Effects of rTMS on a Risky Ball Throwing Task. Journal of the International Neuropsychological Society.

[CR83] Wassermann EM, Zimmermann T (2012). Transcranial magnetic brain stimulation: Therapeutic promises and scientific gaps. Pharmacology & Therapeutics.

[CR84] Weigand A, Grimm S, Astalosch A, Guo JS, Briesemeister BB, Lisanby SH (2013). Lateralized effects of prefrontal repetitive transcranial magnetic stimulation on emotional working memory. Experimental Brain Research.

[CR85] Weissman C, Blumberger D, Brown P, Isserles M, Mulsant B, Downar J (2017). 815. Bilateral repetitive transcranial magnetic stimulation (rTMS) decreases suicidality in adults with treatment resistant depression. Biological Psychiatry.

[CR86] Wölwer W, Lowe A, Brinkmeyer J, Streit M, Habakuck M, Agelink MW (2014). Repetitive Transcranial Magnetic Stimulation (rTMS) Improves Facial Affect Recognition in Schizophrenia. Brain Stimulation.

[CR87] World Health Organization (2017). [Report] Depression and other common mental disorders: Global health estimates. Geneva.

[CR88] World Medical Association Declaration of Helsinki (2013). JAMA.

[CR89] Yang C-C, Khalifa N, Lankappa S, Völlm B (2018). Effects of intermittent theta burst stimulation applied to the left dorsolateral prefrontal cortex on empathy and impulsivity in healthy adult males. Brain and Cognition.

[CR90] Yang C-C, Khalifa N, Völlm B (2018). Excitatory repetitive transcranial magnetic stimulation applied to the right inferior frontal gyrus has no effect on motor or cognitive impulsivity in healthy adults. Behavioural Brain Research.

[CR91] Yazdi K, Rumetshofer T, Gnauer M, Csillag D, Rosenleitner J, Kleiser R (2019). Neurobiological processes during the Cambridge gambling task. Behavioural Brain Research.

[CR92] Zwanzger P, Steinberg C, Rehbein MA, Bröckelmann AK, Dobel C, Zavorotnyy M (2014). Inhibitory repetitive transcranial magnetic stimulation (rTMS) of the dorsolateral prefrontal cortex modulates early affective processing. NeuroImage.

